# Susceptibility of Vegetative Cells and Endospores of *Bacillus cereus* to Rhamnolipid Biosurfactants and Their Potential Application in Dairy

**DOI:** 10.3390/microorganisms10091860

**Published:** 2022-09-17

**Authors:** Paula de Camargo Bertuso, Crisiane Aparecida Marangon, Marcia Nitschke

**Affiliations:** 1 Interunits Graduate Program in Bioengineering (EESC/FMRP/IQSC), University of São Paulo, Trabalhador São-Carlense Av., 400, São Carlos 13566-590, SP, Brazil; 2 Embrapa Instrumentation, Nanotechnology National Laboratory for Agriculture (LNNA), Rua XV de Novembro, 1452, São Carlos 13560-979, SP, Brazil; 3 São Carlos Institute of Chemistry (IQSC), University of São Paulo, Trabalhador São-Carlense Av., 400, P.O. Box 780, São Carlos 13560-970, SP, Brazil

**Keywords:** *Bacillus cereus*, rhamnolipid, endospore, antimicrobial activity

## Abstract

*Bacillus cereus* is a Gram-positive, endospore-forming bacterium well-known as a food pathogen that causes great losses in the food industry, especially in dairy. In this study, rhamnolipid (RL) biosurfactants were evaluated as a bio-based alternative for controlling the growth of vegetative cells and endospores of *B. cereus*. RLs were tested against 14 *B. cereus* strains isolated from different types of foodstuffs. The antimicrobial activity against vegetative cells and endospores revealed minimal inhibitory concentration (MIC) values of 0.098 mg/mL for almost all strains tested and minimal bactericidal concentration (MBC) varying between 0.098 and >25 mg/mL. The presence of RLs inhibited endospore germination by more than 99%, reducing by 5.5 log the outgrowth of strain 0426. Scanning and transmission electron microscopy confirmed that exposure to RL causes damage to the structure of endospores. When skim milk was utilized as a food model, RL inhibited the growth of vegetative cells and endospores of *B. cereus*, showing MBC of 3.13 mg/mL for the vegetative cells of strain 0426. The surfactant also reduced bacterial growth in milk at refrigerator temperature. The results suggest that RLs are promising candidates for the development of novel strategies to control *B. cereus* in the food industry.

## 1. Introduction

According to the World Health Organization, approximately 7.7% of the world’s population falls ill annually due to ingestion of contaminated food, resulting in 420,000 deaths [1]. Epidemiological data show that microbial pathogens are the main cause responsible for the occurrence of outbreaks of foodborne diseases (FBDs). In Brazil, between 2016 and 2019, 2504 outbreaks, 37,247 patients, and 38 deaths related to FBDs were reported, most of them involving bacteria and viruses [2].

*Bacillus cereus*, a rod-shaped, Gram-positive, endospore-forming bacterium, is the fourth most identified etiological agent in outbreaks that occurred in Brazil [2] and the causative agent of several foodborne illnesses reported worldwide. Some recent examples of outbreaks associated with *B. cereus* involve the consumption of beans in the USA [3], beef in Australia [4], and buckwheat in Germany [5]. Due to the ubiquitous nature of *B. cereus*, contamination of both raw materials and finished products can occur and resistance against cleaning treatments applied at the industrial level is increased by their sporulation ability [6]. In fact, *B. cereus* intoxications are facilitated by the presence of endospores, since they can survive cooking and pasteurization processes and may germinate when environmental conditions improve, generating vegetative cells with toxin production capacity [7,8,9].

The control of *B. cereus* is important not only at the public health level but also to avoid economic losses due to food deterioration. Therefore, manufacturers are continuously seeking new methods to guarantee the safety and quality of their products. Currently, there is a great demand for the use of natural products that are safer and do not cause damage to the environment; for this reason, the search for “green” food additives is substantially growing [10]. Moreover, the use of natural products is also interesting considering the development of microbial resistance to the traditionally used disinfectants [11].

Microbial-derived surfactants show high biodegradability, low toxicity, and are synthesized from renewable substrates [12,13]; these characteristics correlate with the principles of green chemistry [14]; thus, such biosurfactants (BSs) represent an important tool for innovation and sustainability, meeting current market demands. Rhamnolipids (RLs) are glycolipid-type BSs predominantly produced by species of the genus *Pseudomonas*. These BSs, in addition to surface and emulsifying activities, also present antimicrobial and antibiofilm actions against various food pathogens [15,16,17] and, due to their versatility, are considered multi-purpose ingredients in food processing [18].

In a recent study, we demonstrated that the presence of RLs promoted a reduction in the population of vegetative cells but could also induce the sporulation of *B. cereus* [19]. In contrast, it was also observed that the germination of endospores could be inhibited by RLs [19]. Although there are some data available in the literature concerning the sensitivity of *B. cereus* to RLs, this was the first report of their activity against endospores of such bacteria. It is known that *B. cereus* endospores present variability in terms of resistance to physical and chemical agents [20,21], and thus it is important to evaluate the sensitivity of strains originating from different foods/environments. Considering the relevance of new sustainable alternatives to control this food pathogen, this work investigates the sensitivity profiles of several food-related strains of *B. cereus* towards rhamnolipid biosurfactants. The potential application of RLs to control the growth of *B. cereus* using milk as a food model is also presented.

## 2. Materials and Methods

### 2.1. Biosurfactant Stock Solution

The rhamnolipid (90% purity) used in this study was acquired from AGAE Technologies (Corvallis, OR, USA). The RL stock solution (50 mg/mL) was prepared in Tryptone Soy Broth (Himedia) supplemented with 6 g/L of yeast extract (TSYEB) and sterilized by filtration (0.22 μm).

### 2.2. B. cereus Strains

*Bacillus cereus* cultures were stored at −20 °C on TSYEB containing 20% (*v*/*v*) glycerol. The strains were mainly isolated from foodstuffs and deposited in different culture collections, such as the American Type Culture Collection (ATCC–USA) and the Culture Collection of Bacillus and Related Genera-Oswaldo Cruz Foundation (CCGB/FIOCRUZ–Brazil). The sources of isolation and the respective collections and codes of the strains are listed below (Table 1).

### 2.3. Fourier-Transform Infrared (FTIR) Spectroscopy

To confirm their identity, RLs were analyzed using Fourier-transform infrared spectroscopy with attenuated total reflectance (ATR-FTIR). The spectrum was obtained from 4000 to 650 cm^−1^, with a resolution of 2 cm^−1^, accumulating 16 scans in a Cary 630 FT-IR spectrometer (Agilent Technologies, Santa Clara, CA, USA).

### 2.4. Determination of Minimal Inhibitory Concentration (MIC) and Minimal Bactericidal Concentration (MBC) of Vegetative Cells and Endospores

In order to test the vegetative cells, stock cultures of *B. cereus* were transferred to a TSEYA (tryptic soy agar supplemented with 6 g/L of yeast extract) plate and incubated at 37 °C for 24 h. The resulting culture was inoculated in 5 mL of TSEYB and maintained at 37 °C for 24 h without agitation. After this period, 1 mL of cell suspension was transferred to 4 mL of fresh TSEYB and incubated for 3 h at 37 °C to guarantee that the inoculum would have mostly vegetative cells. Finally, the suspension optical density (OD) was adjusted to 0.5 at 610 nm (≅3 × 10^7^ CFU/mL).

When the inoculum was prepared with endospores, stock cultures of *B.cereus* were initially streaked in TSEYA and plates incubated for 24 h at 37 °C. Cells were transferred to a modified nutrient agar plate supplemented with 0.06 g/L MgSO_4_ and 0.35 g/L KH_2_PO_4_ to favor sporulation and incubated at 37 °C for 10 days [7]. The resulting culture was suspended in sterilized water and the OD (610 nm) was adjusted to 0.5 (≅3 × 10^6^ CFU/mL). Finally, the suspension was heated to 75 °C for 20 min to eliminate any possible vegetative cells present in the samples [7]. Malachite green staining (Section 2.7) was utilized to confirm sporulation.

An antimicrobial assay was performed on 96-well plates using the micro-broth dilution technique based on Clinical and Laboratory Standards Institute [22] guidelines. Briefly, wells were filled with 100 μL of TSEYB and 100 μL of the RL stock solution was added to the first column following a twofold serial dilution. Next, 20 µL of the standardized bacterial inoculum (10^7^ CFU/mL) was added to each well and the plates were incubated at 37 °C for 24 h. The next day, 10 µL from the wells with no visible growth was plated in TSYEA and incubated at 37 °C for 24–48 h. Further, 20 µL of 0.1% tetrazolium bromide (MTT-Sigma Aldrich) solution was added to the wells and incubated for 1 h to confirm the presence or absence of growth. The MIC was defined as the lowest concentration of RL where no change in the MTT original color was observed; while MBC was defined as the lowest concentration where no cell growth was observed on the agar plate.

### 2.5. Time–Kill Assay

The bacterial growth in the presence of RLs was evaluated for vegetative cells using a time-dependence assay as described by Verma, 2007 [23]. Glass tubes with 5 mL of TSYEB containing RLs at MIC and MBC concentrations were prepared. Inoculum was prepared as described in the previous section and 1 mL of bacterial suspension (OD 0.5) was added to the tubes following incubation at 37 °C without agitation. At pre-determined time intervals, the number of viable cells was assessed after serial dilutions of the samples using the drop method [24]. Tubes with no antimicrobial addition were also assessed as control.

### 2.6. Endospore Germination Inhibition

A total of 1 mL of endospore suspension, obtained as described in Section 2.4, was added to tubes with 5 mL of TSEYB containing pre-determined concentrations (MIC and MBC) of RLs. Samples were incubated at 37 °C without agitation. At specific time points (0, 2, 6, 10, and 24 h), an aliquot from each treatment was taken and a 10-fold serial dilution was performed. Cells were plated on TSYEA using the drop method [24], followed by colony counting after 24 h. Non-treated cells at 0 h were used as control. Log reduction was calculated for each specific time comparatively to the control.

The percentage of endospore germination inhibition and the log reduction were calculated as follows [7]:

(1) $$\%\;\mathrm{endospore}\;\mathrm{germination}\;\mathrm{inhibition}=\frac{\left(CFUcontrol-CFUtreated\right)}{CFUcontrol}\times 100,$$

(2) $$\mathrm{Log}\;\mathrm{reduction}=\log_{10}\left(\frac{CFUcontrol}{CFUtreated}\right)$$

### 2.7. Endospore Staining

A total of 2 mL of cell suspension was centrifuged at 10,000 rpm for 10 min. The supernatant was discarded, and cells were washed with 2 mL distilled water. After a second round of centrifugation, supernatant was discarded and the cell pellet was suspended in 50 μL water. Cells were transferred to a glass slide, heat fixed, and covered with malachite green solution (5%). Slides were passed through a flame for 5 min intermittently, making sure to not boil the dye. The slides were then washed with water and stained with safranin (2.5%) for 30 s [25]. Last, the slides were air dried and observed under a bright field microscope using an immersion lens.

### 2.8. Scanning Electron Microscopy (SEM) of B. cereus Endospores

Sample preparation for SEM analysis was performed as described by MURTEY; RAMASAMY, 2016 [26] with adaptations. Endospores were prepared as described in Section 2.4, inoculated in culture media with and without RL, and incubated at 37 °C for a pre-determined time (0, 2, and 24 h). After incubation, samples were centrifuged at 10,000 rpm for 10 min. The supernatant was discarded and the pellet was washed twice with saline solution (NaCl 0.86%). Finally, the pellet was suspended in 20 μL of saline solution and transferred to circular glass slides (13 mm diameter) previously coated with poly-lysine (poly-lysine hydrobromide type III, Sigma) and dried in room temperature under laminar flow. The slides were fixed over 90 min in 3% glutaraldehyde solution prepared in phosphate buffer (pH 7.2). After fixation, the slides were washed with buffer and dehydrated in ethanol solution with a crescent concentration gradient (35, 50, 75, 95, and 2× 100%) for 10 min each. Next, samples were submerged in hexamethyldisilazane (HMDS, Sigma) until the time they received the gold coating. The samples were analyzed in a scanning electron microscope LEO 440 (Zeiss) operating at 15 kV.

### 2.9. Transmission Electron Microscopy (TEM) of B. cereus Endospores

Endospores were prepared as described previously (Section 2.4), inoculated in culture media with RL, and incubated at 37 °C for 24 h. Next, samples were centrifuged at 10,000 rpm for 10 min. The supernatant was discarded and the pellet was washed twice with saline solution (NaCl 0.86%). Following this, the samples were resuspended in 400 μL of 2.5% glutaraldehyde solution prepared in sodium cacodylate buffer 0.1 M (pH 7.2) and incubated overnight at room temperature. The samples were centrifuged at 10,000 rpm for 10 min, suspended in 500 μL of sodium cacodylate buffer 0.1 M, and stored in a refrigerator until the time for processing [27]. Samples were embedded in 2% noble agar, followed by a post-fixation step with 2% osmium tetroxide for 1 h at 4 °C and dehydratation in a graded series of ethanol. Endospores were embedded in EPON 812 resin at 60 °C and ultrathin sections (thickness of 50 nm) were double-stained with uranyl acetate and lead [28]. Microscopy was performed on a JEM 100 CXII TEM (JEOL) operated at 80 kV acceleration voltage.

### 2.10. Growth of Vegetative Cells and Endospores of B. cereus in Skim Milk

To study the antimicrobial effect of RL in a food model, skim powdered milk reconstituted at 10% concentration was utilized. The milk sample was sterilized in an autoclave at 121 °C for 10 min one day prior to use. Using milk as culture medium, MIC and MBC tests were performed as described in Section 2.4. Vegetative cells and endospores were inoculated, incubated at 30 °C, and diluted to an initial population of approximately 1 × 10^4^ CFU/mL. Endospore presence was confirmed by staining, as described above. Once the values corresponding to the MIC/MBC were found, growth curves at 30 °C and 4 °C [29] were constructed to study the performance of RL against the bacteria in milk. The experiment set up consisted of mixing 5 parts of milk with or without RL to 1 part of inoculum. When vegetative cells were inoculated, the incubation was undertaken at 4 °C, and samples were taken at different time-intervals and submitted to serial dilution and viable counts. When endospores were inoculated, the incubation was done either at 4 °C or at 30 °C and samples were separated in two sets. One was submitted to serial dilution and total viable counts (endospores + vegetative cells) and the other was heated at 75 °C for 20 min, serially diluted, and plated in TSYEA in order to eliminate the vegetative cells and perform only the endospore count [29].

### 2.11. Statistics

Values for MIC and MBC are expressed as the mode of at least three independent experiments. The remaining data represent the means ± standard deviations (SD) of three independent experiments.

## 3. Results and Discussion

### 3.1. Fourier-Transform Infrared (FTIR) Spectroscopy

**Figure 1:**
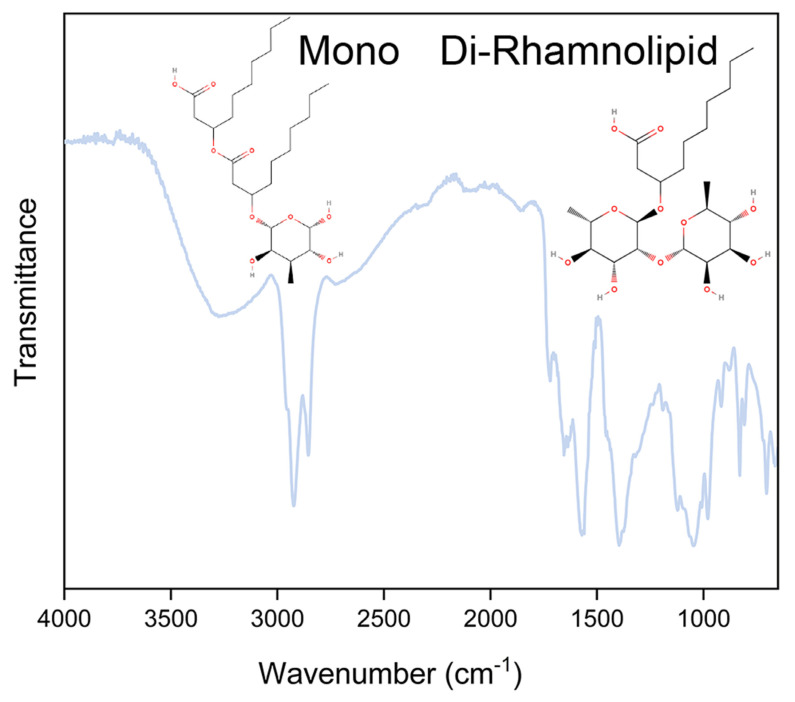
ATR-FTIR spectrum of rhamnolipid and its chemical structures.

Due to the rhamnolipid microbial origin, several factors may influence its features, such as the type of rhamnolipid and the distribution of di-rhamnolipid and mono-rhamnolipid homologs present in the mixture, which depends on the strain used, carbon source, extraction process, and other factors [30]. Furthermore, variations in the sugar moiety, carbon chain type, and length of rhamnolipids are known to affect their biological activity [31]. Rhamnolipids produced by *P. aeruginosa* are often composed of one or two rhamnose molecules bound to one or two hydroxyl fatty acids. An ATR-FTIR spectrum (Figure 1) enabled identifying the functional groups present in the rhamnolipid used in this study. The characteristic absorption bands at 3272 cm^−1^ corresponded to stretching vibrations of hydroxyl groups (OH). The sharp bands at 2925 and 2851 cm^−1^ were expected to be C−H bands of the hydrocarbon chain. Absorption at 1722 cm^−1^ represented the presence of the carbonyl group (−C=O) of esters and that at 1571–1397 cm^−1^ (−C=O) the presence of carboxylic acid [32]. The characteristic bands at 1317–982 cm^−1^ confirmed the presence of –C–OH formed between carbon atoms and hydroxyl groups in the chemical structures of the rhamnose rings [33].

Tests performed by our research group (data not shown) using high performance liquid chromatography mass spectrometry (HPLC-MS) have also shown that the antimicrobial used in this study is composed of mono- and di-rhamnolipids, with mono-rhamnolipid homologs representing 80% of the composition.

### 3.2. Determination of Minimal Inhibitory Concentration (MIC) and Minimal Bactericidal Concentration (MBC)

*B. cereus* strains used in this study were treated with RLs with the aim of determining the minimal concentrations needed to inhibit or eliminate the vegetative cells and endospores of the bacteria. As shown in Table 2, the predominant MIC value for the RLs was 0.098 mg/mL. In contrast, it was possible to observe a great variation in MBC concentrations for both vegetative cells and endospores. Vegetative cells of the 11778, 33018, and 0524 strains and endospores of the 33018 and 0426 strains showed no MBC within the tested concentrations, suggesting that, for them, RLs were bacteriostatic. It is also worth noting that, even when considering strains isolated from the same type of food, such as 33018, 33019, 0534, and 1734, all originating from powdered milk, the differences in MBC were still evidenced, with values ranging from 0.098 to 12.5 mg/mL.

The antimicrobial action of RLs against *B. cereus* cells has been described in the literature; in contrast, their activity against endospores was only recently reported [19]. The similar MIC values observed for both vegetative cells and endospores, as well as the lower MBC for some endospores compared to vegetative cells (Table 2), were not expected since the spores are resistance structures, which can tolerate unfavorable growing conditions and different environmental stress factors [9,34]. Although studies using RLs against *B. cereus* or other endospore-forming bacteria are not available, some reports with other antimicrobials have also found similar results. Ávila et al. (2014) [35] compared the activity of nisin, reuterin, lysozyme, and sodium nitrite against vegetative cells and endospores of *Clostridium* spp. The authors observed the same MIC values for vegetative and endospores against all the compounds tested but, in general, the spores were more resistant; in addition, antimicrobial effectiveness varied widely among the *Clostridium* species and was influenced by the bacterial form and by the culture medium. The MIC of nisin against *Clostridium difficile* was 5.20 mg/L and 3.47 mg/L for vegetative cells and endospores, respectively [36], an example of the highest sensitivity to spores.

Few reports in the literature compare MBC results for vegetative cells and endospores, and most antimicrobials are in fact considered sporostatic rather than sporicidal. Sensitivity tests of *Bacillus thuringiensis* subsp. *israelensis* (Bti) and subsp. *kurstaki* (Btk) and *Bacillus subtilis* ATCC 6051 against several antibiotics showed that metabolically active vegetative cells were two times more resistant to antibiotics than spores [37]. These data illustrate that the susceptibility of endospores may, in some cases, be higher than that of vegetative cells. In this study, the MBC of endospores was checked after 24 and 48 h, as well as longer times; however, it is also possible that the RL treatment induced superdormant spores not able to germinate even in the presence of nutritionally and environmentally optimal conditions, retarding their growth in culture medium and affecting MBC results.

Further studies were conducted to confirm the observed activity of RLs against endospore and vegetative *B. cereus* cells.

### 3.3. Time–Kill Assay and Endospore Germination Inhibition

Strain 0426 was chosen as a model for subsequent experiments aiming to evaluate bacterial (vegetative and endospore) growth in culture medium and milk.

**Figure 2:**
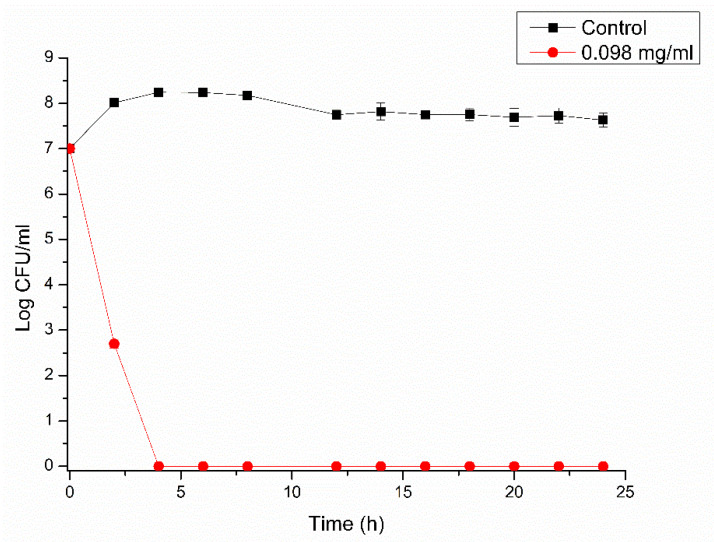
Growth behavior of *Bacillus cereus* 0426 vegetative cells in the presence of RL (0.098 mg/mL) compared to the control. Data represent the means ± SD of three independent experiments.

In Figure 2, it is possible to observe that the concentration of 0.098 mg/mL was able to completely eliminate the vegetative cells within 4 h of exposure to RL, corroborating the data shown in Table 2.

**Figure 3:**
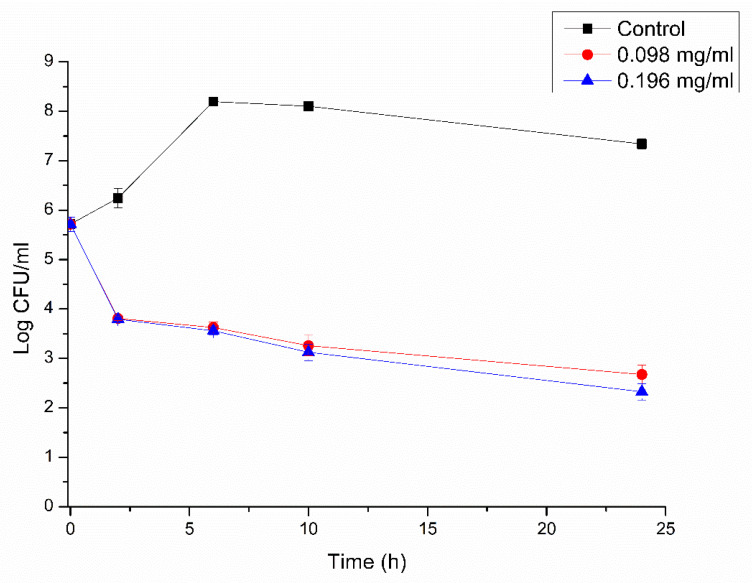
*Bacillus cereus* 0426 endospores outgrowth in the presence of RLs. Values of the MBC (0.098 mg/mL) and 2× MBC (0.196 mg/mL) for vegetative cells were utilized. Data represent the means ± SD of three independent experiments.

Endospores were treated with RLs with different times of exposure and the percentage of germination inhibition and log reduction were calculated. After 2 h of exposure, more than 99% of endospore germination was inhibited using 0.098 mg/mL of RLs. The increment of RL concentration (2×) and time of exposure (24 h) did not significantly increase germination inhibition (data not shown). Furthermore, a log reduction of 5.5 was observed after 24 h treatment with RLs compared to the control (Figure 3). Even for *B. cereus* strains where an MBC was not found, RLs showed a great capacity for inhibition of endospore germination, with values up to 93% inhibition [19]. A similar report using mannosylerythritol lipid (MEL) biosurfactants showed endospore germination inhibition up to 98% with concentrations of 1.25 mg/mL [38].

### 3.4. Electron Microscopy of B. cereus Endospores

**Figure 4:**
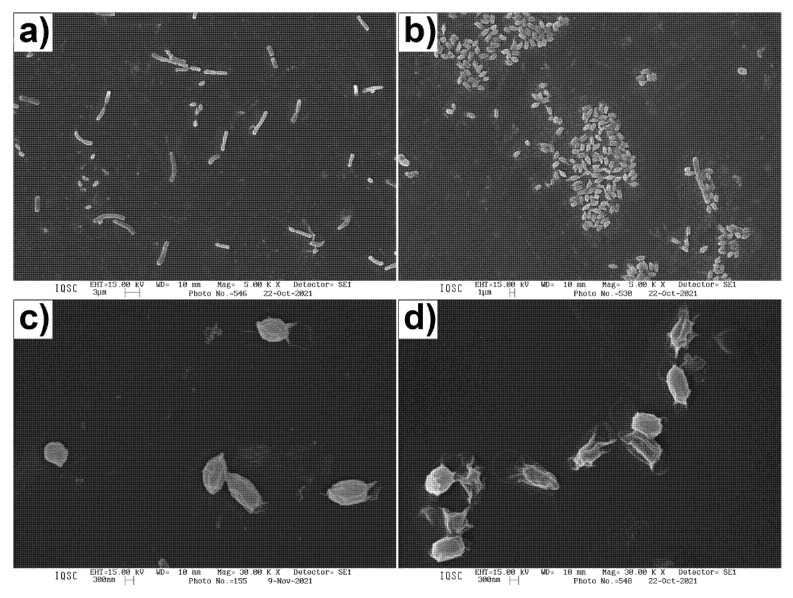
SEM images of *B. cereus* 0426 endospores: (**a**) 2 h growth control, (**b**) 24 h growth with 0.098 mg/mL of RL, (**c**) control endospore at 0 h, and (**d**) RL-treated endospores after 24 h.

**Figure 5:**
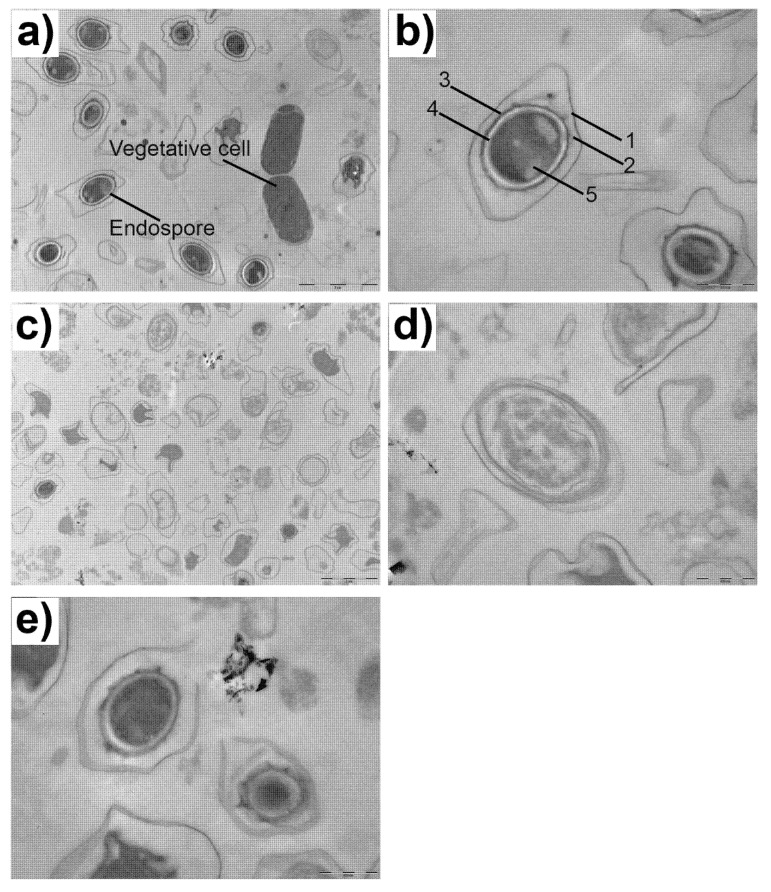
TEM images of *B. cereus* 0426 endospores: (**a**) control showing a vegetative cell and intact endospores; (**b**) details of endospore structures: (1) exosporium, (2) spore coat, (3) outer membrane, (4) cortex, and (5) core containing DNA and ribosomes; (**c**) treatment with RLs (0.098 mg/mL) for 24 h; (**d**) close-up of an endospore with internal structure damaged and (**e**) exosporium disrupted. Magnification bars represent 2 µm (**a**,**c**) or 500 nm (**b**,**d**,**e**).

To demonstrate the effect promoted by RL treatment in endospore structure, the samples were visualized using scanning and transmission electron microscopy (Figure 4 and Figure 5).

Figure 4a shows that, 2 h after being transferred to media without the antimicrobial component, the majority of spores were capable of germinating. This observation is consistent with other reports in the literature using the same bacteria [39,40]. However, super-dormant populations that may take several hours or even days to germinate were also described [40,41]. This fact could help explain the presence of some endospores in Figure 4a.

Figure 4b, on the other hand, shows that the treatment with RLs for 24 h inhibited endospore germination. These images corroborate the data presented in Table 2 and Figure 3, since the concentration used for this test was the MIC value obtained for the endospores of this strain. Moreover, even this concentration was not able to completely eliminate endospores; the presence of RLs at the concentration of 0.098 mg/mL prevented germination and, thus, could inhibit bacterial contamination and further toxin production. Control samples (Figure 4c) showed intact endospores with a relatively rough surface and exosporium. However, when endospores were treated with RLs for 24 h, they appeared to be misshapen with a wrinkled exosporium, and some spores seemed to have suffered lysis (Figure 4d).

Although the damages caused by RL in the endospore morphology could have been related to the loss in their capacity for germination and, consequently, their ability to cause disease, there are reports in the literature showing endospore inactivity even without visible structural modifications [42,43]. For this reason, the combination of different image techniques is necessary.

TEM was performed to observe how the treatment with RLs could affect endospores’ internal structure. Figure 5a,b show intact endospores with preserved structures, while Figure 5c–e show damaged endospores after treatment with RLs for 24 h. It is interesting to notice that, when comparing Figure 5d,e, it is possible to observe differences in damage extension for different endospores. Figure 5d shows an endospore with a relatively intact exosporium, an internal structure without defined coats, and heterogeneous electron density. In contrast, Figure 5e shows a disruption in the exosporium with a relatively preserved internal structure.

Green tea polyphenols were also reported to change the surface of *B. cereus* endospores from smooth to more irregular and, in some cases, with cell lysis [7]. The authors suggested that the integrity alterations in the endospores promoted by the treatment would be responsible for the high rates of germination inhibition observed. TEM images revealed that the tested polyphenols were capable of destroying endospores coats, which compromises the structural stability, and are likely to be responsible for inhibiting endospore germination [7].

In a similar way, wrinkling on the external surface of *B. cereus* endospores was observed after moist heat treatment (105 °C for 60 s) [43]. Other groups have also shown the capacity of heat to inactivate and promote morphology alterations, such as wrinkling of the external layer or even the presence of disruptions and holes in the surface of *B. cereus* endospores [44].

A lipopeptide produced by *Bacillus subtilis*, which showed activity against *B. cereus,* also promoted morphological changes in endospores. The treated spores showed undefined structures, blurry coats, coarse surfaces, and even damaged coats that led to leakage of cellular content [28].

The inhibition promoted by RL for *B. cereus* vegetative cells is relative to their surfactant nature since they can interfere with cytoplasmic membrane integrity and cause cell disruption, leakage of nutrients, and consequent cell death [45,46,47]. Differently from vegetative cells, endospores have several protective barriers that enhance their resistance. The exosporium, spore coats, inner and outer membranes, cortex, and core (Figure 5b) are targets for interaction with chemical agents [34]. The activity of amphiphilic compounds has also been described against endospores, as they can interact with several groups present in spore coats, including their membranes. Ceragenin, a bile acid derivative, effectively inactivated *B. subtilis* endospores by affecting the integrity of inner membrane; a consequent loss of Ca-DPA was detected and the disruption of spores was attributed to the permeabilizing effect of the surfactant [48]. The surface of the spores is predominantly hydrophobic; thus, more apolar compounds can easily interact with them [49]. In addition, the HBL of the surfactants has been correlated with endospore inactivation potential [50]. In agreement with these statements, we can hypothesize that RL surfactant affects endospores integrity (as illustrated by SEM and TEM images) and, therefore, their germination and outgrowth. Further studies should be conducted to elucidate the mechanistic aspects of the RL activity.

### 3.5. Evaluation of Growth of Vegetative Cells and Endospores of B. cereus in Skim Milk

The antimicrobial potential of RL in controlling *B. cereus* using skim milk as a food model was evaluated to study its applicability at the industrial level.

First, MBC tests were performed for vegetative cells and endospores of strain 0426 at 30 °C. The MIC could not be determined because the MTT solution also shows color alteration when added to milk, making it difficult to determine the concentrations where bacterial growth was not observed. For vegetative cells, an MBC of 3.13 mg/mL was found for RLs, while for endospores, the MBC was not observed within the concentrations tested (MBC > 50 mg/mL).

When compared to the data presented in Table 2 for strain 0426, there was a significant difference between MBC values for vegetative cells grown in TSYEB (0.098 mg/mL) and in milk (3.13 mg/mL). A hypothesis for this difference lies in the complexity of the milk matrix when compared to the culture media; as the presence of proteins and minerals may make the interaction of the surfactant with the cells difficult, protecting them from its action. Other groups have also studied the protective effects of milk components for bacteria subjected to high pressure and ultrasound treatments [51,52]. For treatment with high pressure, samples containing milk showed surviving values around 2 log higher than that observed for samples containing phosphate buffer. The authors also discussed which milk components may be responsible for this difference in survival and found that phosphocasein, together with phosphate, citrate, calcium, and magnesium minerals, were able to protect *Listeria innocua* cells from the harsh treatment [51]. In another study, most of the *Clostridium* spp. strains (vegetative and endospores) were found to be more resistant to antimicrobials in milk than in culture medium [35]. Another hypothesis is that RL behavior may be altered by the presence of milk constituents, as suggested in previous work [15], considering that the antimicrobial activity of RL is dependent on several factors, including pH, temperature, nutrients, and micellar structures [15,45,53]. Moreover, it is important to consider that the bacterial cells or spores growing in milk might have different cell surface properties compared to the ones grown in the culture medium, and such characteristics can influence their sensitivity to the surfactant [54].

**Figure 6:**
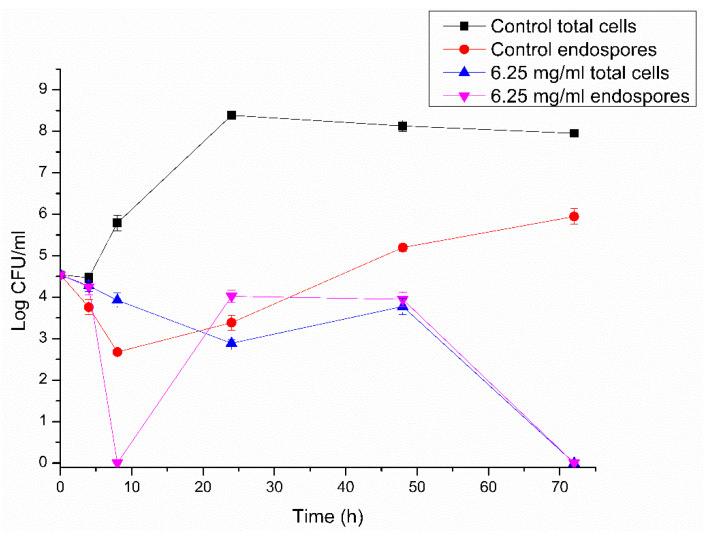
Growth of *B. cereus* 0426 endospores in skim milk at 30 °C in the presence of RLs (6.25 mg/mL). Data represent the means ± SD of three independent experiments.

Even without an MBC value for endospores, a growth curve experiment at 30 °C was performed with an inoculum containing endospores and using the concentration of the 2× MBC of vegetative cells (6.25 mg/mL) to evaluate whether the presence of RLs in milk was also able to inhibit germination (Figure 6).

Figure 6 shows that, for the control sample, during the first 4 h, the number of endospores began to drop significantly while the values for total cells (endospores + vegetative cells) remained relatively stable. The fast germination observed in the control can also be seen in Figure 4a, where after only 2 h in culture media it was possible to observe high amounts of vegetative cells.

It is also worth noting that, over time, the total number of cells in the control stabilized while an increase in endospore population was observed, suggesting that the cells may have been responding to limitations imposed by culture conditions or by the media itself.

For samples containing 6.25 mg/mL of RL, the population, both for total cells and endospores, did not increase beyond the initial number. In addition, when RLs were present, the growth cultures showed a similar tendency, illustrating the surfactant ability to inhibit endospore germination and also induce cell eradication after 72 h.

Aiming to study the effect of RL in storage conditions, the behavior of vegetative cells and endospores of *B. cereus* 0426 was evaluated under refrigeration temperature (4 °C).

**Figure 7:**
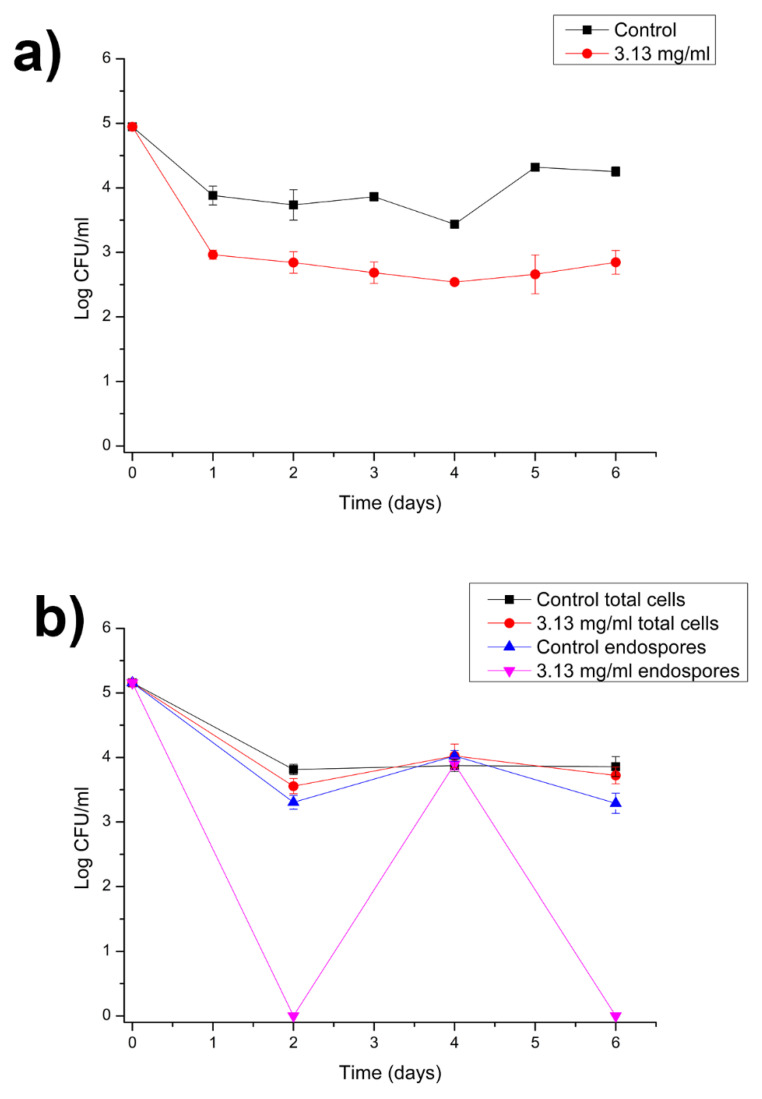
Growth of *B. cereus* 0426 in skim milk at 4 °C in the presence of RLs (3.13 mg/mL). (**a**) Inoculum of vegetative cells and total colony counts and (**b**) Inoculum of endospores and colony counts of both vegetative cells and endospores. Data represent the means ± SD of three independent experiments.

Figure 7a (inoculum with vegetative cells) shows that, on the first day, a drop in viable cells was observed for all tested samples, with 1 log drop for the control and approximately 2 log drops for the treatment. It is important to consider the temperature a limiting factor for cell growth, as a significant population growth was not observed over the 6 days of testing. This observation is consistent with literature data that indicated that *B. cereus* may grow in temperatures ranging from 4 to 48 °C [55]. Thus, cells can be expected to grow at a lower rate when exposed to low temperatures.

Figure 7b (inoculum with endospores) shows the behavior of endospores in storage conditions. Initially, it was possible to observe a decrease in total cell population for both control and RL treatments, followed by relative stability throughout the days of the experiment. When observing the endospore count, on the other hand, it was noticeable that, when RLs were present, the count dropped substantially in the first days, reaching nearly zero colonies, followed by an increase in endospore counts to nearly the total counts of the treated sample and then a decrease to values close to zero again. This curve suggests that, during storage time, cells transit between their endospore and vegetative form.

When evaluating the effect of temperature on *B. cereus* growth in refrigerated vegetable subtracts, VALERO; FERNÁNDEZ; SALMERÓN, 2003 [56] reported that, from the six lineages tested, only one showed bacterial growth at 8 °C, while all lineages were capable of growing when stored at 12 °C. More recently, other research groups have also reported that, at 8 °C, the lineage ATCC 33019 did not show any growth for either vegetative cells or endospores of *B. cereus* [57].

Observing Figure 7, it is also possible to conclude that, with the decrease in the temperature, the values once found to be bactericidal for vegetative cells became bacteriostatic. The reason for this may have been the fact that low temperatures may induce sporulation, making cells more resistant to RL action. Nevertheless, samples containing RL showed lower cell survival, illustrating RL potential in controlling bacterial growth in milk substrate.

The population of emerging vegetative cells growing in milk seems to be more sensitive to the surfactant; thus, while some cells died, others sporulated, and this repeating cycle could explain the eradication of the population observed after 72 h at 30 °C (Figure 6). At low temperature, in four days, the total cell counts represented spore counts, and their further germination increased the population of vegetative cells that persisted in milk after six days. The presence of RLs visibly enhanced sporulation/germination cycles compared to the control. We can speculate that the population (Figure 7b) may gradually be reduced, showing a similar behavior as seen in Figure 6 but with prolonged times due to the low temperature.

The antimicrobial efficacy of a fungal glycolipid against *Bacillus weihenstephanensis* and other psychrotolerant spore-forming bacteria was evaluated in UHT whole and skim milk at 7 °C for 3 weeks. Results showed that the presence of the glycolipid was able to delay growth of the spoilage bacteria in milk, thus increasing its shelf life [29]. A combination of nisin with refrigeration (6 °C) was tested to hinder *B. cereus* growth in milk for 21 days, being capable of controlling the growth of vegetative cells and germination of endospores [58]. Other glycolipids, such as mannosylerythritol lipid-A (MEL-A), were used to control *B. cereus* in frozen bread dough, eliminating 99.97% of vegetative cells and 75.54% of endospores after a simulated dough fermentation process [59].

The distribution and size of RL aggregates was determined at 10, 25, and 37 °C, demonstrating that, as the temperature increased, RL micelle size also tend to increase [15]. This changing in micelle size under different temperatures may have influenced RL solubilization in media [15,60], which could have affected their availability to interact with cells and, consequently, their antimicrobial action. This fact could also help to explain why a bactericidal concentration at 30 °C became bacteriostatic at 4 °C (Figure 7a) and why the presence of RLs may lead cells to germinate and then sporulate again.

In summary, our results suggest that RLs can control the growth of *B. cereus*, both vegetative and spores, showing potential for application in the dairy industry and in the development of novel strategies and hurdle technologies to guarantee the safety of food products.

## Figures and Tables

**Table 1:** Origin of the *Bacillus cereus* strains utilized in this study.

Code	Collection	Isolated From
10876	ATCC	Contaminated flask
11778	ATCC	Not informed
33018	ATCC	Infant milk formula
33019	ATCC	Infant milk formula
0426	CCGB/FIOCRUZ	Chicken pot pie
0477	CCGB/FIOCRUZ	Milk
0524	CCGB/FIOCRUZ	Raw beef
0534	CCGB/FIOCRUZ	Skim powdered milk
0547	CCGB/FIOCRUZ	Cooked corn meal
1217	CCGB/FIOCRUZ	Powdered nutmeg
1223	CCGB/FIOCRUZ	Oregano
1224	CCGB/FIOCRUZ	Raw Yuca flour
1517	CCGB/FIOCRUZ	Coffee
1734	CCGB/FIOCRUZ	Powdered milk

**Table 2:** Antimicrobial activity of RLs against vegetative cells and endospores of *B. cereus*.

	Vegetative Cells (mg/mL)		Endospores (mg/mL)	
Strain Code	MIC	MBC	MIC	MBC
10876	0.098	6.25	0.098	0.78
11778	0.78	>25	0.098	1.56
33018	0.098	>25	0.098	>25
33019	0.098	0.098	0.098	3.13
0426	0.098	0.098	0.098	>25
0477	0.098	0.098	0.098	6.25
0524	0.098	>25	0.098	6.25
0534	0.098	0.098	0.098	1.56
0547	0.098	0.098	0.098	3.13
1217	0.098	6.25	0.098	6.25
1223	0.098	6.25	0.098	1.56
1224	0.098	25	0.098	3.13
1517	0.098	0.098	0.098	25
1734	0.098	12.5	≥0.049	6.25

## Data Availability

The data presented in this study are available in the article.
